# Supplementary material for: shRNA-mediated down-regulation of Acsl1 reverses skeletal muscle insulin resistance in obese C57BL6/J mice
Source: PLoS One. 2024 Aug 23;19(8):e0307802. doi: 10.1371/journal.pone.0307802 (PMC11343424; doi:10.1371/journal.pone.0307802)
Supplement: S1 Appendix — (PDF) [file pone.0307802.s001.pdf]

S1 Appendix

shRNA-mediated down-regulation of Acsl1 reverses skeletal muscle insulin resistance in obese C57BL6/J mice

Kamila Roszczyc-Owsiejczuk [1], Monika Imierska[1], Emilia Sokołowska[1], Mariusz Kuźmicki[2], Karolina Pogodzińska [1], Agnieszka Błachnio-Zabielska [1*¶] and Piotr Zabielski [3*¶]

[1]Department of Hygiene, Epidemiology and Metabolic Disorders, Medical University of Bialystok, Bialystok, Poland

[2]Department of Gynecology and Gynecological Oncology, Medical University of Bialystok, Bialystok, Poland

[3]Department of Medical Biology, Medical University of Bialystok, Bialystok, Poland

***Corresponding authors:**

Piotr Zabielski, PhD, DSc,

Email: piotr.zabielski@umb.edu.pl

Agnieszka Blachnio-Zabielska, PhD, Prof.

email: agnieszka.blachnio-zabielska@umb.edu.pl

[¶] These authors contributed equally to this work.

Materials and Methods

Sample size calculation

The size of the experimental groups was determined based on the results of previous experiments conducted in C57BL/6 mice fed a high-fat diet, and a review of recent data from other research teams using C57BL6/J mice for insulin resistance studies. The analysis of the power and size of the groups was based on the variability of the HOMA-IR parameter, muscle content of diacylglycerol and ceramide. The analysis showed that the optimal size of the experimental groups for $p<0.05$ and 90% power is 6 for the HOMA-IR parameter, 8 for muscle lipids. Analysis of the literature showed that the size of the experimental group ranged from 6 to 20 subjects per group in the case of studies using diet-induced insulin resistance. Therefore, the optimal number of individuals in the group was set at 8.

Randomization

All inclusion and exclusion criteria was described in approval 35/2016. Animals were randomized, using a computer based random order generator. On arrival from Jackson Laboratory (Bar Harbor, ME, USA), mice were weighed and assigned to random cages. Each animal was assigned a temporary random number. On the basis of their position on the rack, cages were given a numerical designation. After the acclimatization period, the cages were randomized within the exposure group.

Animal care and monitoring

In accordance with the principle of refinement, the experimental procedures were appropriately modified in order to reduce the suffering and stress of the animals while ensuring the acquisition of high-quality data. To reduce the pain of collecting a blood drop for glucose assessment, the tail of the mouse was locally anesthetized with lidocaine. The similar treatment was applied to the site of electrode placement directly after electroporation. Electroporation procedure was performed under isoflurane anesthesia. In order to reduce postoperative muscle soreness during the first 24 hours, the animals received appropriate analgesic in water (ibuprofen, 0.5mg/ml). During the experiment we do not observe any expected or unexpected adverse events and the electroporation procedure was well tolerated, with normal behavior returning after 24 hours after the procedure.

Any of the symptoms listed below were not observed: no activity either spontaneous or induced; distress - vocalization independent of contact with other individuals or the researcher; severe respiratory rhythm disturbances; prolonged shaking; prolonged seizures (>10min); paralysis and/or drugged state (lasting more than 30 minutes); diarrhea; weight loss >20% of animal's last weight prior to weight loss classified for immediate euthanasia. During the experiment, the above-mentioned symptoms were absent.

Intramuscular delivery of shRNA plasmid

Briefly, mice were placed in an induction chamber. Anesthesia was induced by administering ~2% isoflurane in oxygen with a flow rate of 0.750 l/min. Once anesthetized, the rodents were placed on a

heating blanket and gaseous anesthesia was maintained by administering ~2% isoflurane at a flow rate of 0.4 l/min using a respiratory pump for small animals (UNO BV rodent anesthesia system UNO, Zevenaar, the Netherlands). After complete sedation, the area above the gastrocnemius muscle was shaved and washed with sterile 0.9% NaCl solution. Then an intramuscular injection of hyaluronidase was performed in two doses of 15 µl (0.4 U/µL hyaluronidase in sterile Tyrode). After 120 minutes animals received intramuscular injection of 40 µl of plasmid suspension (from 2 µg/µL stock) using a 27-gauge needle. Electroporation current (8 pulses, 200ms each, 175 V/cm^2) was applied immediately after injection through a pair of silver electrode plates (area of 1 cm^2, electrodes distance 4mm) with the use of BTX ECM 830 Electroporation Generator pulse generator (Holliston, MA, USA).

Electroporation-based plasmid transfection of mouse hindlimb muscle – visualization of TurboGFP reporter.

Photographs of the gastrocnemius muscles were taken using a DeltaPix Invenio 5SIII CMOS camera. Fluorescence microscopy was obtained by a Nightsea SFA-RB-GO fluorescence adapter upon exciting tissue fluorescence with a ~480-nm light source (Nightsea, Andover, USA).

Insulin-stimulated glucose uptake

Insulin-stimulated glucose uptake was assessed to observe the effects of Acsl1 silencing on skeletal muscle insulin sensitivity in high-fat diet fed animals. Briefly, 20 minutes prior to euthanasia, a bolus of radiolabeled glucose was injected into the lateral tail vein (bolus of 0.5 µCi/g animal, Perkin-Elmer, Waltham, MA), followed by 0.5 U of insulin (NovoRapid, Novo Nordisk A/S, Bagsværd, Denmark) per kg of fat-free mass, administered intraperitoneally. Afterwards, blood samples were collected at 2.5, 5, 7.5, 10, 12.5 and 15 minutes into the heparinized tube. Plasma was immediately separated by centrifugation and stored at -80°C until analysis. Additionally, during the collection of blood samples, plasma glucose was measured by means of an Accu-Chek Aviva glucometer (Roche, Mannheim, Germany). After deproteinization with $Ba(OH)_2$ (0.3 N) and $ZnSO_4$ (0.3 N), 2-deoxy-[1,2-3H (N)]-D-glucose radioactivity in plasma and tissue was determined by liquid scintillation counting with Packard Tri Carb 1900TR liquid scintillation counter (Perkin Elmer, Waltham, MA). Skeletal muscle samples were weighed and sonicated in 6% $HClO_4$ to release tissue-incorporated radioactive tracer. Homogenates were centrifuged and neutralized with 5 M of KOH. Subsequently, samples were treated with 0.3 N $Ba(OH)_2$/0.3 N $ZnSO_4$ to fractionate 2-deoxy D-glucose (tracer) and 2-deoxy-d-glucose-6-phosphate. In all of the experiments, the tissue-incorporated 2-deoxyglucose-6-phosphate was calculated as difference in radioactivity in $HClO_4$ and $Ba(OH)_2$/$ZnSO_4$ tissue extracts.

Insulin-stimulated glucose uptake was calculated using the following equation:

$$Rg = \frac{Cm * (T)}{\int_0^T (Cp^* / Cp)} * \Delta t$$

where Cm* (T) is the tissue radioactivity of 2-deoxy-[1,2-3H (N)]-d-glucose-6-phosphate/mg at the end of the experiment (DPM/mg), Δt is the time from glucose bolus injection to tissue collection (min) and $\int$T,0 (Cp∗/Cp) is the area under the plasma glucose enrichment curve calculated with the use of the trapezoidal rule (DPM/mg/min).

Plasma FFA

The concentration of plasma FFA was measured to establish the impact of high-fat diet on the plasma concentration of free fatty acids which is one of the factors responsible for lipid-induced skeletal muscle insulin resistance. In short, Dole extraction mixture was freshly prepared by mixing isopropanol: heptane: 1 M H_2SO_4 (40:10:1; v/v/v). The internal standard (C14:0-d27, C15:0, C16:0-d31, C17:0, C18:1-d9 and C18:0-d35; Avanti Polar Lipids, Alabaster, AL, USA) and extraction mixture were added to each plasma sample. The concentrations of FFA were measured against a six-point standard curve prepared with albumin-conjugated FFA chemical standards. The mixture was centrifuged. The supernatant was transferred into a new tube and evaporated under nitrogen. The dried samples were reconstituted in LC/MS solvent A, vortexed and transferred into new tubes for analysis. The analytical column was a reverse-phase ZORBAX SB-C18 column 2.1 x 150 mm, 1.8 μm (Agilent Technologies, Santa Clara, CA, USA). Chromatographic separation was conducted with the use of a two-buffer system: 80% acetonitrile and 0.5 mM ammonium acetate as solvent A; and 99% acetonitrile and 1% 0.5 mM ammonium acetate as solvent B. The concentrations of FFA were measured against a six-point standard curve prepared with albumin-conjugated FFA chemical standards.

LCACoA and SCACoA

We measured skeletal muscle LCACoA and SCACoA concentration to observe the effects of Acsl1 silencing and high-fat diet feeding on the muscular activation of free fatty acids. A particular amount of internal standard (C15:0-CoA, 16:0(d4) CoA, C17:0-CoA, C19:0-CoA, C21:0-CoA, C23:0-CoA and 24:0(d4) CoA; Avanti Polar Lipids, Alabaster, AL, USA) was added prior to the extraction. The following compounds: C2:0-CoA, C3:0-CoA, C4:0-CoA, C14:0-CoA, C16:0-CoA, C16:1-CoA, C18:2-CoA, C18:1-CoA, C18:0-CoA, C20:0-CoA, C22:0-CoA, C24:1-CoA and C24:0-CoA (Avanti Polar Lipids, Alabaster, AL, USA) were separated on a reverse-phase Agilent ZORBAX Extend-C18 Column, 2.1 x 150 mm, using a binary gradient with ammonium hydroxide (NH_4OH) in water and NH_4OH in ACN. The analysis and quantification of LCACoA was performed on a triple quadrupole mass spectrometer (Sciex QTRAP 6500+, AB Sciex Germany GmbH, Darmstadt, Germany) in positive electrospray ionization (ESI) mode against the concentration standard curves prepared for each compound. Values were normalized to initial sample weight and expressed in pmol/mg of tissue.

Acyl-carnitine

The content of acyl-carnitine molecular species was measured to asses mitochondrial transport of activated fatty acids in HFD-fed animals under the Acsl1 silencing. Gastrocnemius sample pulverizate

was extracted with the use of ice-cold methanol. After centrifugation aqueous phase was dried under nitrogen stream and acyl-carnitines were derivatized with 100 µl of n-butanol/5% acetyl chloride (v/v) to butyl esters for 20 minutes at 60°C with shaking. Samples were evaporated to dryness, reconstituted in 100 µl methanol/water and transferred to autosampler vials for further analysis. Acyl-carnitines were analyzed with the use of Sciex QTRAP 6500+ triple quadrupole mass spectrometer (AB Sciex Germany GmbH, Darmstadt, Germany) in +ESI ionization with MRM mode. The chromatographic separation was performed by UHPLC on reverse-phase ZORBAX SB-C18 column (2.1 x 150 mm, 1.8 µm, Agilent Technologies, Santa Clara, CA, USA) with the use of Shimadzu Nexera-X2 UHPLC. Tissue concentration of acyl-carnitines was measured against standard curves constructed for each compound. The resulting values were normalized to original tissue weight and expressed in pmol/mg of tissue.

Sphingolipids

Sphingolipids are implicated in the induction of skeletal muscle insulin resistance under fatty acids overload. Briefly, gastrocnemius samples (~20 mg) were pulverized under liquid nitrogen and then homogenized in a solution composed of 0.25 M sucrose, 25 mM KCl, 50 mM Tris and 0.5 mM EDTA, pH 7.4, with a set amount of deuterated internal standards (Sph-d7, SPA-d7, S1P-d7, C15:0-d7-Cer, C16:0 -d7-Cer, C18:1-d7-Cer, C18:0-d7-Cer, 17C/20:0-Cer, C24:1-d7-Cer and C24-d7-Cer, Avanti Polar Lipids, Alabaster, AL, USA). Immediately afterwards, an extraction mixture (isopropanol: water: ethyl acetate, 30:10:60; v/v/v) was added to each sample. The mixture was vortexed, sonicated and then centrifuged for 10 minutes at 3000 g. The supernatant was transferred into a new tube and the pellet was reextracted. After centrifugation, supernatants were combined and evaporated under nitrogen. The dried samples were reconstituted in LC solvent B (2 mM ammonium formate, 0.1% formic acid in methanol), vortexed and transferred into a new tube for analysis. The sphingolipids were analyzed by means of a Sciex QTRAP 6500+ triple quadrupole mass spectrometer (AB Sciex Germany GmbH, Darmstadt, Germany) using a positive ion electrospray ionization (ESI) source (except for S1P, which was analyzed in the negative mode) with multiple reaction monitoring (MRM) against standard curves constructed for each compound. Chromatographic separation was performed with the use of an ultraperformance liquid chromatograph (Shimadzu Nexera X2 UHPLC, Shimadzu Corporation, Kyoto, Japan). The analytical column was a reverse-phase ZORBAX SB-C8 column 2.1 x 150 mm, 1.8 µm (Agilent Technologies, Santa Clara, CA, USA). Chromatographic separation was conducted in binary gradient with the use of 1 mM ammonium formate, 0.1% formic acid in water as solvent A, and 2 mM ammonium formate and 0.1% formic acid in methanol as solvent B at the flow rate of 0.4 mL/min. Resulting values were normalized to original sample weight and expressed in pmol/mg of tissue.

Diacylglycerols

Immediately prior to extraction of diacylglycerols and sphingolipids, internal standard mix (Deuterated DAG Mixture I and Mixture II, Avanti Polar Lipids, Alabaster, AL, USA) was added to skeletal muscle samples. The samples were extracted using identical procedure as described below. The following DAG:

C18:1/18:2, C16:0/18:2, C16:0/16:0, C16:0/18:1, C18:0/20:0, C18:0/18:1, C18:1/18:1, C18:0/18:2 and C16:0/18:0 were analyzed on Sciex QTRAP 6500+ MS/MS (AB Sciex Germany GmbH, Darmstadt, Germany) in +ESI ionization using MRM mode. Content of tissue DAG was calculated against the concentration standard curves prepared for each compound. Resulting values were normalized to original sample weight and expressed in pmol/mg of tissue.

Western blot

Briefly, muscle samples were homogenized in RIPA buffer (Merck KGaA, Darmstadt, Germany) supplemented with 0.5 mM tris(2-carboxyethyl)phosphine (TCEP, reducing agent, Merck KGaA, Darmstadt, Germany), protease inhibitor cocktail (cOmplete™ ULTRA Tablets, Roche) and phosphatase inhibitor cocktail (PhosSTOP™, Roche). After centrifugation at 10,000 × g at 4°C for 30 minutes, supernatant was collected and protein content was measured using a Pierce 660 nm protein assay kit (Thermo Fisher Scientific, Waltham, MA, U.S.). After denaturation and reduction in Laemmli sample buffer proteins of interest were separated with SDS-PAGE on AnykD Criterion TGX gels and transferred to a polyvinylidene difluoride (PVDF) membrane using BioRad Trans Blot SD semidry transfer cell and discontinuous buffer system; (Tris/CAPS/15% methanol for anode and Tris/CAPS 0.1% SDS for cathode). After blocking and washing, the membrane was incubated at 4°C overnight with primary antibodies (list of antibody are presented in Table S2. Blots were incubated with appropriate HRP-conjugated secondary antibody and protein bands were visualized and quantified by chemiluminescence using a Clarity Western ECL chemiluminescent substrate (Bio-Rad), Bio-Rad ChemiDoc XRS+ imaging system and Bio-Rad Image Lab software package (Software version 6.1). Values were normalized to GAPDH protein expression measured from parallel runs and expressed as fold changes over control group values. All chemicals and equipment used for immunoblotting were purchased from Bio-Rad.
