# Supplementary material for: shRNA-mediated down-regulation of Acsl1 reverses skeletal muscle insulin resistance in obese C57BL6/J mice
Source: PLoS One. 2024 Aug 23;19(8):e0307802. doi: 10.1371/journal.pone.0307802 (PMC11343424; doi:10.1371/journal.pone.0307802)
Supplement: S1 Fig — Study was performed on the gastrocnemius muscle of C57BL/6J mice. (PDF) [file pone.0307802.s002.pdf]

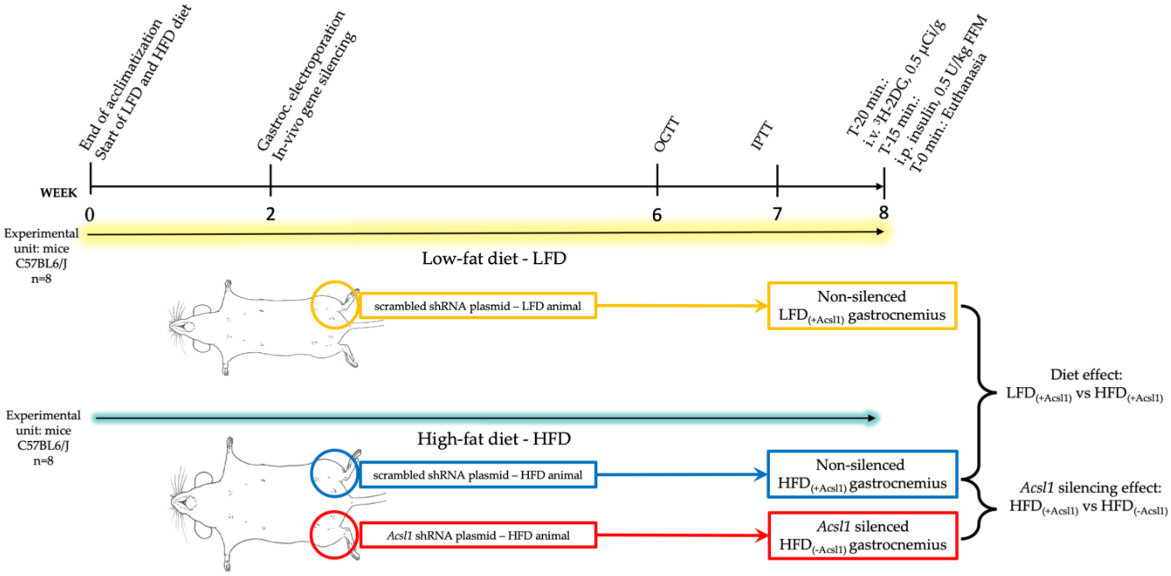

S1 Figure. Description of the experiment design.

Study was performed on the gastrocnemius muscle of C57BL/6J mice. Low-fat diet mice (LFD) received scrambled shRNA plasmid, yielding non-silenced LFD$_{(+Acsl1)}$ tissue with intact Acsl1 expression. Right hindlimb gastrocnemius of high-fat diet mice (HFD) was electroporated with scrambled shRNA, yielding non-silenced HFD$_{(+Acsl1)}$ gastrocnemius. The contralateral, left gastrocnemius muscle within the same animal was transfected with active shRNA plasmid, yielding silenced HFD$_{(-Acsl1)}$ gastrocnemius. Non-silenced tissue from LFD and HFD animals was compared to study the effect of HFD diet (LFD$_{(+Acsl1)}$ vs HFD$_{(+Acsl1)}$ muscle). Silenced and non-silenced tissue from HFD animals was compared to study the effects of Acsl1 silencing (HFD$_{(+Acsl1)}$ vs HFD$_{(-Acsl1)}$ muscle).
