# Supplementary material for: shRNA-mediated down-regulation of Acsl1 reverses skeletal muscle insulin resistance in obese C57BL6/J mice
Source: PLoS One. 2024 Aug 23;19(8):e0307802. doi: 10.1371/journal.pone.0307802 (PMC11343424; doi:10.1371/journal.pone.0307802)
Supplement: S2 Fig — (PDF) [file pone.0307802.s003.pdf]

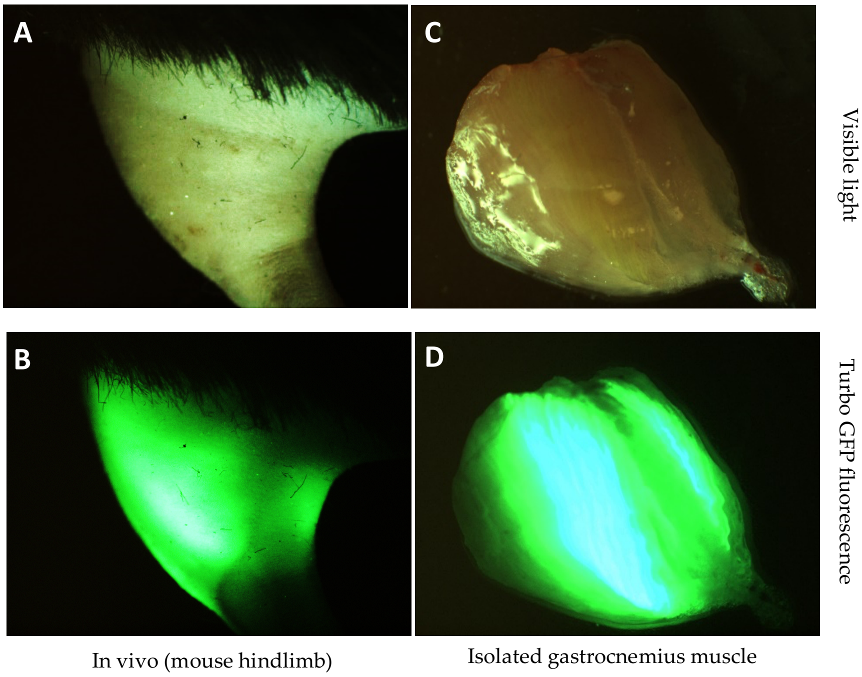

S2 Figure. Visualization of green fluorescent protein (TurboGFP) reporter gene expression in mouse hindlimb at 6 weeks after electroporation-mediated plasmid transfection.

Panel **(A)**—visible light photo of a mouse left hindlimb; Panel **(B)**—transcutaneous TurboGFP fluorescence of electroporated muscle; Panel **(C)**—visible light photo of an isolated mouse gastrocnemius muscle; Panel **(D)**—TurboGFP fluorescence of an electroporated gastrocnemius muscle. Visualization performed with Nightsea SFA-RB-GO fluorescence adapter/DeltaPix Invenio 5SIII CMOS camera.
