# Supplementary material for: shRNA-mediated down-regulation of Acsl1 reverses skeletal muscle insulin resistance in obese C57BL6/J mice
Source: PLoS One. 2024 Aug 23;19(8):e0307802. doi: 10.1371/journal.pone.0307802 (PMC11343424; doi:10.1371/journal.pone.0307802)
Supplement: S3 Fig — (PDF) [file pone.0307802.s004.pdf]

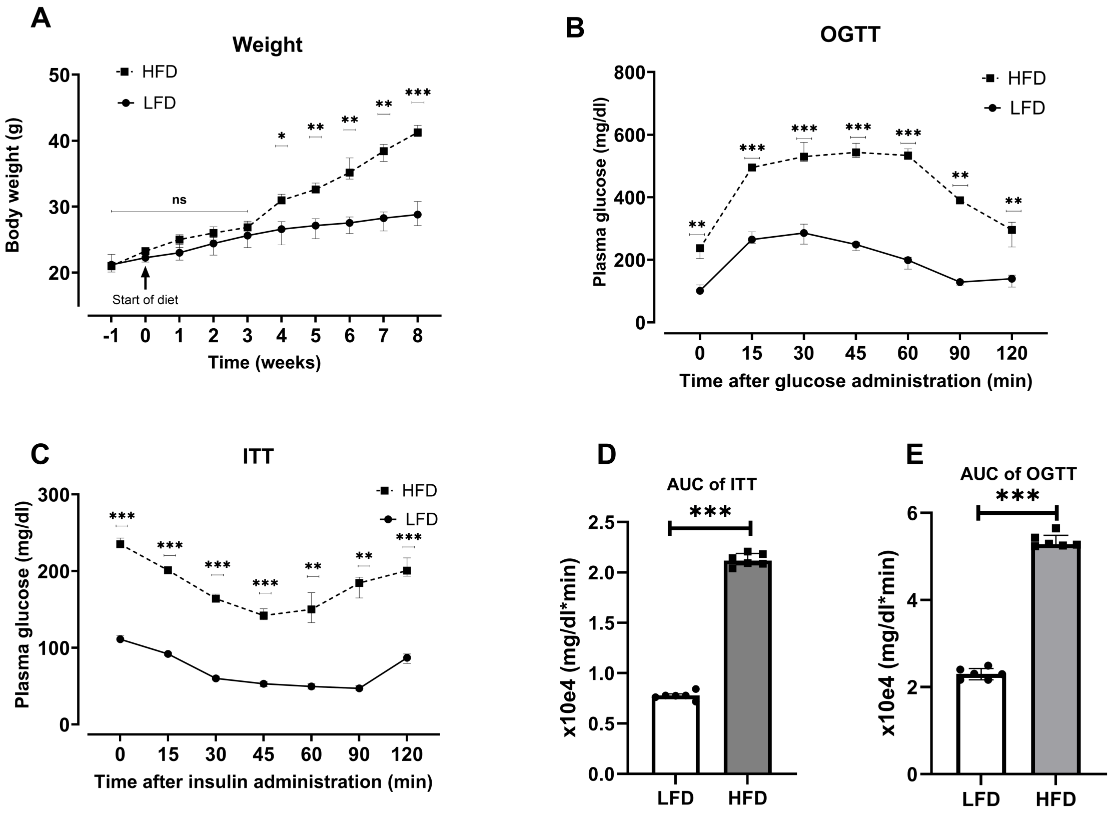

S3 Figure. Characteristics of HFD-induced obesity and insulin resistance in C57BL/6J mice.

Panel A—body weight curves; Panel B —plasma glucose profile during oral glucose tolerance test (OGTT); Panel C—plasma glucose levels during intraperitoneal insulin tolerance test (ITT); Panel D—area under the plasma glucose curve for OGTT; Panel E—area under the plasma glucose curve for ITT. The area under the curve (AUC) was calculated using the trapezoidal rule. LFD – mice fed low-fat diet; HFD – HFD-fed mice. Values are median ± interquartile range (n = 8 per group); Statistics by Wilcoxon rank sum test for non-paired samples; ^{ns} -p > 0.05; * - p ≤ 0.05; **- p ≤ 0.01; ***- p≤0.001 vs LFD values.
