# Supplementary material for: shRNA-mediated down-regulation of Acsl1 reverses skeletal muscle insulin resistance in obese C57BL6/J mice
Source: PLoS One. 2024 Aug 23;19(8):e0307802. doi: 10.1371/journal.pone.0307802 (PMC11343424; doi:10.1371/journal.pone.0307802)
Supplement: S4 Fig — (PDF) [file pone.0307802.s005.pdf]

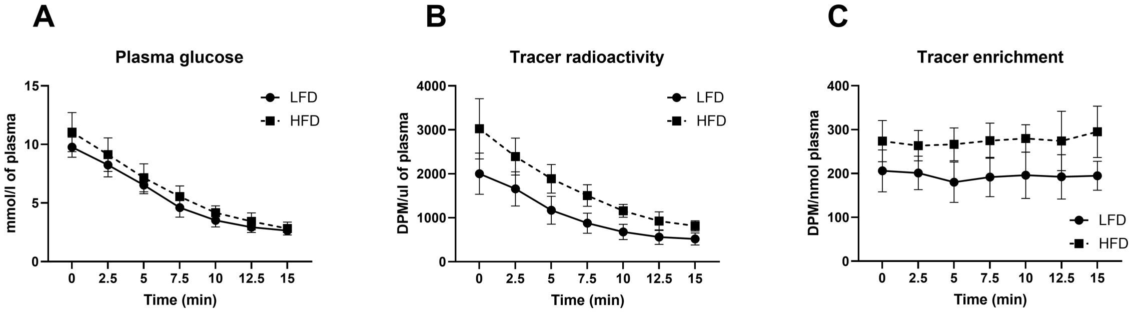

S4 Figure. Plasma glucose and 2-deoxy-[1,2-3H (N)]-D-glucose profiles during 0.5 U/kg intraperitoneal insulin challenge.

Panel (**A**)—plasma glucose concentration; Panel (**B**)—plasma 2-deoxy [1,2-3H (N)]-d-glucose tracer radioactivity; Panel (**C**)—plasma glucose tracer enrichment after intravascular tracer and insulin bolus injection. Values are median and interquartile range; n = 8 per group. LFD – mice fed low-fat diet; HFD – HFD- mice fed high-fat diet.
