# Supplementary material for: shRNA-mediated down-regulation of Acsl1 reverses skeletal muscle insulin resistance in obese C57BL6/J mice
Source: PLoS One. 2024 Aug 23;19(8):e0307802. doi: 10.1371/journal.pone.0307802 (PMC11343424; doi:10.1371/journal.pone.0307802)
Supplement: S5 Fig — (PDF) [file pone.0307802.s006.pdf]

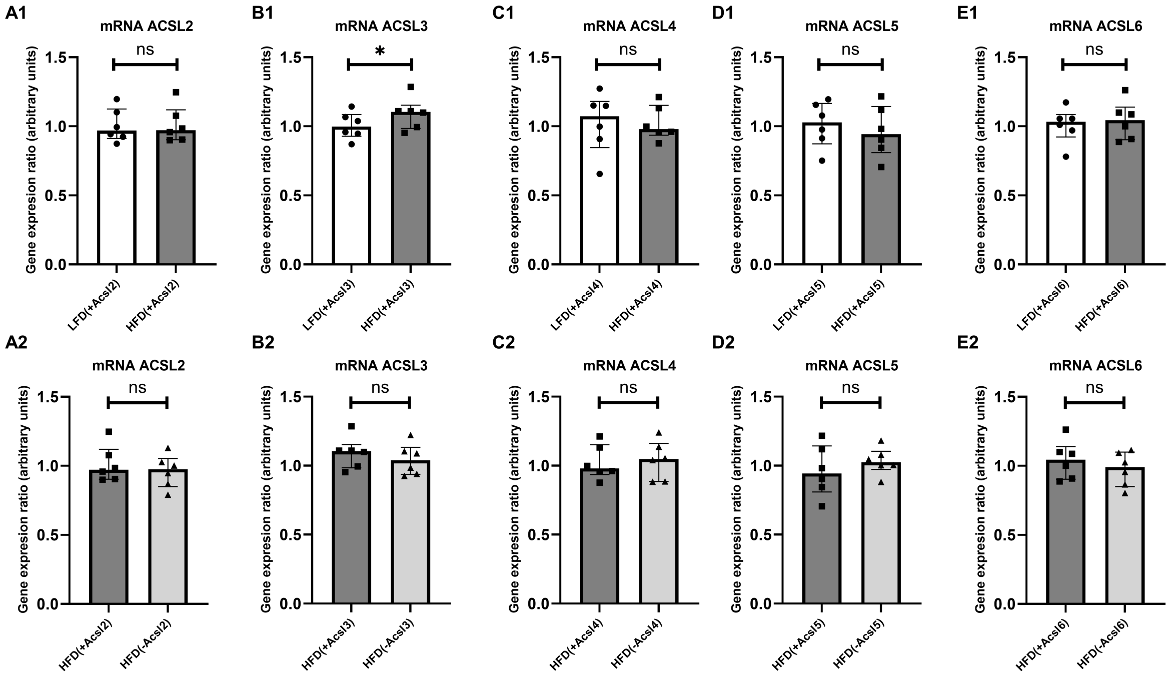

S5 Figure. The impact of high-fat diet and Acsl1 silencing on the gene expression of other skeletal muscle acyl-CoA synthetase isoforms.

Panels A1 to E1 present effect of diet ($LFD_{(+Acsl1)}$ and $HFD_{(+Acsl1)}$ muscle). Panels A2 to E2 present effect of Acsl1 silencing within HFD-fed animals ($HFD_{(+Acsl1)}$ vs $HFD_{(-Acsl1)}$ muscle). $LFD_{(+Acsl1)}$ – gastrocnemius from LFD-fed mice, with intact Acsl1 expression (scrambled plasmid); $HFD_{(+Acsl1)}$ - gastrocnemius from HFD-fed mice with intact Acsl1 expression (scrambled plasmid); $HFD_{(-Acsl1)}$ – contralateral hindlimb gastrocnemius from HFD-fed mice, with down-regulated Acsl1 expression (silencing shRNA plasmid). Values are median ± interquartile range; n=6 per group. ns – $p > 0.05$; * – $p \leq 0.05$.
