# Supplementary material for: shRNA-mediated down-regulation of Acsl1 reverses skeletal muscle insulin resistance in obese C57BL6/J mice
Source: PLoS One. 2024 Aug 23;19(8):e0307802. doi: 10.1371/journal.pone.0307802 (PMC11343424; doi:10.1371/journal.pone.0307802)
Supplement: S1 Table — (PDF) [file pone.0307802.s007.pdf]

S1 Table. List of the antibodies used in the study.

Cat. No	Antibody	Company
ab33780	GLUT4	Abcam
NB400-144	CD36	Novus Biologicals
H00376497-D01P	FATP1	Novus Biologicals
ab153924	FABPpm	Abcam
ab104662	CPT1	Abcam
PA553317	HADHA	Thermo Fisher Scientific
PA582655	ACADVL	Thermo Fisher Scientific
PA595971	ACADM	Thermo Fisher Scientific
4047	ACSL1	Cell Signaling Technology
3662	ACC	Cell Signaling Technology
3661	pACC(Ser76)	Cell Signaling Technology
4691S	Akt	Cell Signaling Technology
4060P	pAktSer473	Cell Signaling Technology
2965S	pAktThr308	Cell Signaling Technology
2390	IRS1	Cell Signaling Technology
sc-17196	pIRS1(Y632)	Santa Cruz Biotechnology
2385	pIRS1(Ser1101)	Cell Signaling Technology
ab5500	IR	Abcam
ab5678	pIR(Y972)	Abcam
2670S	AS160	Cell Signaling Technology
8730S	AS160(Ser588)	Cell Signaling Technology
ab9485	GAPDH	Abcam
