# Supplementary material for: shRNA-mediated down-regulation of Acsl1 reverses skeletal muscle insulin resistance in obese C57BL6/J mice
Source: PLoS One. 2024 Aug 23;19(8):e0307802. doi: 10.1371/journal.pone.0307802 (PMC11343424; doi:10.1371/journal.pone.0307802)
Supplement: S2 Table — (PDF) [file pone.0307802.s008.pdf]

S2 Table. Sequence of the primers used in the study.

Acsl1	R - TAAGTAAGGCAGTGTTCCGT<br>F - ACTGTGCAGGAACAAGGATAT
Acsl2	R - CCGTACCAGTCAAGGTGTGC<br>F - CGAGGATTTTTCGAGGACGC
Acsl3	R - CGTTGTTGCAGAAAGCGGAG<br>F - CTCCACTGTGTGACCGCTTT
Acsl4	R - CCCTCAGGGTACTCTGCTCT<br>F - GGAAAGCAAACTGAAGGCGG
Acsl5	R - TGAGCCAAGGGAAGGTAGGA<br>F - CAGTGGAACTACAGGTGACCC
Acsl6	R - GCCTGGTGGAAGATCTTGTCA<br>F - TGTTGTCTACTGCCACGGAG
GAPDH	R - AGGAGACAACCTGGTCCTCA<br>F - AGGAGAGTGTTTCCTCGTCC
