# Supplementary material for: shRNA-mediated down-regulation of Acsl1 reverses skeletal muscle insulin resistance in obese C57BL6/J mice
Source: PLoS One. 2024 Aug 23;19(8):e0307802. doi: 10.1371/journal.pone.0307802 (PMC11343424; doi:10.1371/journal.pone.0307802)
Supplement: S3 Table — (PDF) [file pone.0307802.s009.pdf]

S3 Table. Plasma, blood morphology parameters and concentration of individual plasma free fatty acids of the LFD-fed and HFD-fed mice. LFD – mice fed low-fat diet; HFD – HFD- mice fed high-fat diet.

	LFD	HFD
**Fasting glucose (mg/dL)**	109(100-124)	181(166-200)*
**Fasting insulin (ng/ml)**	0.78(0.66-0.88)	1.70(1.61-1.76)*
**HOMA-IR**	1.16(1.13-1.20)	3.13(2.81-3.56)*
**FFA (nmol/ml)**	257.2(253.3-273.3)	509.7(498.8-516.7)*
**TAG (ng/ul)**	119.1(108.4-141.3)	228.1(223.0-250.6)*
**WBC ($10^3/mm^3$)**	7.1(6.3-7.5)	4.3(4.1-4.6)*
**RBC ($10^4/mm^3$)**	11.0(10.6-11.7)	10.5(10.0-11.4)*
**HGB (g/dL)**	15.3(14.0-15.7)	14.8(13.5-15.6)*
**HCT (%)**	54.9(51.6-54.9)	51.9(48.7-56.3)*
**MCV (fl)**	49.0(49.0-49.3)	49.0(49.0-50.0)*
**MCH (pg)**	13.6(13.2-13.9)	13.9(13.5-14.1)*
**MCHC (g/dL)**	27.7(26.9-28.0)	28.0(27.6-28.3)*
**PLT ($10^3/mm^3$)**	270.0(216.5-324.0)	866.5(739.0-923.0)*
**C14:0 FFA**	7.62(5.59-7.95)	17.22(16.48-17.57)*
**C16:0 FFA**	69.44(64.61-74.81)	96.74(88.30-101.50)*
**C16:1 FFA**	5.18(5.09-8.05)	5.30(5.07-5.73)*
**C18:0 FFA**	29.01(27.19-30.81)	61.43(58.93-63.63)*
**C18:1 FFA**	66.38(61.75-74.09)	141.50(133.70-146.50)*
**C18:2 FFA**	54.47(50.74-69.66)	140.40(135.80-144.40)*
**C20:0 FFA**	1.14(0.84-1.33)	4.04(3.66-4.37)*
**C20:2 FFA**	2.37(1.97-2.78)	2.48(2.30-2.65)*
**C20:4 FFA**	10.13(8.27-11.03)	19.38(18.77-20.22)*
**C22:0 FFA**	3.95(3.60-4.28)	11.01(10.85-11.98)*
**C22:6 FFA**	1.68(1.56-1.82)	2.13(2.04-2.28)*
**C24:0 FFA**	3.49(3.20-4.76)	7.93(7.43-8.37)*
**C24:1 FFA**	0.17(0.14-0.18)	0.39(0.31-0.42)*

Values median and interquartile range (n = 8 per group). Statistics by Wilcoxon rank sum test for non-paired samples; * -p ≤ 0.05 vs LFD values.
