# Supplementary material for: shRNA-mediated down-regulation of Acsl1 reverses skeletal muscle insulin resistance in obese C57BL6/J mice
Source: PLoS One. 2024 Aug 23;19(8):e0307802. doi: 10.1371/journal.pone.0307802 (PMC11343424; doi:10.1371/journal.pone.0307802)
Supplement: S4 Table — (PDF) [file pone.0307802.s010.pdf]

S4 Table. The effect of a high-fat diet on the content of individual lipid in mouse gastrocnemius. $LFD_{(+Acsl1)}$ – gastrocnemius from low-fat diet fed C57BL/6J mice, transfected with scrambled shRNA plasmid; $HFD_{(+Acsl1)}$ – gastrocnemius from high-fat diet fed C57BL/6J mice transfected with scrambled shRNA plasmid.

		$LFD_{(+Acsl1)}$	$HFD_{(+Acsl1)}$
**Short- and long-chain acyl-CoA**	**C2:0**	1.18(1.00-1.30)	1.60(1.51-1.87)**
	**C3:0**	19.06(15.44-23.41)	31.68(25.78-34.91)**
	**C4:0**	0.017(0.013-0.018)	0.029(0.026-0.030)**
	**C14:0**	0.12(0.10-0.13)	0.20(0.17-0.22)**
	**C16:0**	0.82(0.78-0.95)	0.92(0.90-1.11) **
	**C16:1**	0.76(0.66-0.82)	0.69(0.61-0.73) **
	**C18:0**	0.73(0.66-0.82)	1.18(1.07-1.34)**
	**C18:1**	2.59(2.28-2.81)	2.74(2.26-3.13) **
	**C18:2**	1.71(1.45-1.98)	2.04(1.74-2.18) **
	**C20:0**	0.016(0.015-0.017)	0.018(0.016-0.022) **
	**C22:0**	0.022(0.019-0.023)	0.031(0.029-0.036) **
	**C24:0**	0.029(0.024-0.031)	0.031(0.030-0.034) **
	**C24:1**	0.015(0.013-0.020)	0.008(0.008-0.010)**
**Short- and long-chain acyl-carnitines**	**C2**	54.21(46.86-64.07)	48.92(44.75-56.87)**
	**C3:0**	3.08(2.09-3.51)	3.81(3.38-4.03)**
	**C4**	4.21(3.86-5.10)	3.73(3.49-4.13) **
	**C5**	2.61(2.37-3.06)	2.18(1.96-2.73) **
	**C5:0-DC**	0.017(0.015-0.020)	0.017(0.016-0.020) **
	**C6**	0.82(0.77-0.85)	0.91(0.86-1.05) **
	**C8**	0.30(0.29-0.36)	0.21(0.18-0.23)**
	**C10**	0.23(0.20-0.26)	0.18(0.15-0.21)**
	**C12**	0.32(0.28-0.36)	0.29(0.26-0.34) **
	**C14**	1.51(1.32-1.63)	1.26(1.03-1.33)**
	**C16**	2.45(2.26-2.57)	2.94(2.71-3.11) **
	**C18:1**	6.98(6.41-7.29)	7.46(6.80-7.67) **
	**C18**	0.74(0.62-0.78)	1.15(1.13-1.40)**
**Diacylglycerol**	**16:0/16:0**	13.10(11.99-14.29)	16.23(13.75-18.29) **
	**16:0/18:0**	82.52(72.56-93.61)	138.70(134.20-162.70)**
	**16:0/18:1**	33.36(28.28-36.33)	39.97(37.53-42.99)**
	**16:0/18:2**	39.55(33.12-42-59)	65.92(55.17-76.33)**
	**18:0/18:0**	2.11(1.88-2.28)	2.89(2.74-3.17)**
	**18:0/18:1**	21.31(19.68-24.27)	22.62(21.11-26.33) **
	**18:0/18:2**	0.98(0.80-1.12)	1.98(1.76-2.18)**
	**18:1/18:1**	20.40(18.96-23.35)	23.55(21.49-25.05) **
	**18:2/18:2**	24.74(22.95-26.71)	33.88(28.67-39.18)**
	**18:0/20:0**	3.89(3.70-3.93)	7.03(6.58-8.05)**
**Sphingolipid**	**d18:1/C14:0**	0.029(0.028-0.031)	0.034(0.027-0.038) **
	**d18:1/C16:0**	1.935(1.843-2.131)	3.290(2.848-3.477)**
	**d18:1/C18:0**	17.18(15.36-18.20)	29.91(28.12-33.03)**
	**d18:1/C18:1**	0.565(0.486-0.635)	0.667(0.613-0.710)**
	**d18:1/C20:0**	0.276(0.253-0.300)	0.571(0.469-0.645)**
	**d18:1/C22:0**	0.758(0.708-0.852)	1.442(1.294-1.596)**
	**d18:1/C24:0**	1.252(1.166-1.357)	2.574(2.377-2.648)**
	**d18:1/C24:1**	3.780(3.488-4.203)	5.063(4.532-5.385)**

Values show median (pmol/mg of tissue) and interquartile range ; n=8 per group; Statistics by Wilcoxon rank sum test for non-paired samples; ^{ns} -p > 0.05; * -p ≤ 0.05; **- p ≤ 0.01 vs $LFD_{(+Acsl1)}$.
