# Supplementary material for: shRNA-mediated down-regulation of Acsl1 reverses skeletal muscle insulin resistance in obese C57BL6/J mice
Source: PLoS One. 2024 Aug 23;19(8):e0307802. doi: 10.1371/journal.pone.0307802 (PMC11343424; doi:10.1371/journal.pone.0307802)
Supplement: S5 Table — (PDF) [file pone.0307802.s011.pdf]

S5 Table. The effect of *in vivo* shRNA-mediated Acsl1 gene down-regulation on the content of individual lipid in mouse gastrocnemius in high-fat diet mice. $HFD_{(+Acsl1)}$ – gastrocnemius from high-fat diet fed C57BL/6J mice transfected with scrambled shRNA plasmid; $HFD_{(-Acsl1)}$ – contralateral hindlimb gastrocnemius, transfected with Acsl1 silencing shRNA plasmid.

		$HFD_{(+Acsl1)}$	$HFD_{(-Acsl1)}$
**Short- and long-chain acyl-CoA**	C2:0	1.60(1.51-1.87)	1.40(1.35-1.48) *
	C3:0	31.68(25.78-34.91)	17.98(16.09-19.46)**
	C4:0	0.029(0.026-0.030)	0.018(0.016-0.020)**
	C14:0	0.20(0.17-0.22)	0.15(0.14-0.17)**
	C16:0	0.92(0.90-1.11)	0.68(0.62-0.74) **
	C16:1	0.69(0.61-0.73)	0.41(0.36-0.45) **
	C18:0	1.18(1.07-1.34)	0.79(0.65-0.81)**
	C18:1	2.74(2.26-3.13)	1.44(1.25-1.70)**
	C18:2	2.04(1.74-2.18)	1.37(1.21-1.44)**
	C20:0	0.018(0.016-0.022)	0.013(0.013-0.017) **
	C22:0	0.031(0.029-0.036)	0.018(0.017-0.020)**
	C24:0	0.031(0.030-0.034)	0.017(0.015-0.019)**
	C24:1	0.008(0.008-0.010)	0.009(0.008-0.010)ns

**Short- and long-chain acyl-carnitines**	C2	48.92(44.75-56.87)	31.21(27.62-34.41)**
	C3:0	3.81(3.38-4.03)	1.86(1.72-2.16)**
	C4	3.73(3.49-4.13)	1.21(1.10-1.26)**
	C5	2.18(1.96-2.73)	1.28(1.07-1.30)**
	C5:0-DC	0.017(0.016-0.020)	0.018(0.015-0.021)ns
	C6	0.91(0.86-1.05)	0.19(0.17-1.05)**
	C8	0.21(0.18-0.23)	0.03(0.03-0.03) **
	C10	0.18(0.15-0.21)	0.06(0.06-0.07)**
	C12	0.29(0.26-0.34)	0.13(0.11-0.15)**
	C14	1.26(1.03-1.33)	0.46(0.42-0.52)**
	C16	2.94(2.71-3.11)	1.28(1.21-1.37)**
	C18:1	7.46(6.80-7.67)	2.69(2.48-3.21)**
	C18	1.15(1.13-1.40)	0.46(0.39-0.53)**

**Diacylglycerol**	C16:0/16:0	16.23(13.75-18.29)	12.02(10.75-15.01)**
	C16:0/18:0	138.70(134.20-162.70)	64.35(53.96-71.41)**
	C16:0/18:1	39.97(37.53-42.99)	24.91(24.50-26.98)**
	C16:0/18:2	65.92(55.17-76.33)	21.75(19.43-24.29)**
	C18:0/18:0	2.89(2.74-3.17)	0.98(0.85-1.12)**
	C18:0/18:1	22.62(21.11-26.33)	18.22(16.45-20.25)**
	C18:0/18:2	1.98(1.76-2.18)	1.82(1.69-1.94)**
	C18:1/18:1	23.55(21.49-25.05)	16.46(15.15-18.35)**
	C18:2/18:2	33.88(28.67-39.18)	18.60(17.45-21.29)**
	C18:0/20:0	7.03(6.58-8.05)	4.56(3.50-5.51)**

**Ceramide**	d18:1/C14:0	0.034(0.027-0.038)	0.025(0.024-0.028)**
	d18:1/C16:0	3.290(2.848-3.477)	2.139(1.911-2.554)**
	d18:1/C18:0	29.91(28.12-33.03)	22.21(21.45-23.90)**
	d18:1/C18:1	0.667(0.613-0.710)	0.337(0.299-0.358)**
	d18:1/C20:0	0.571(0.469-0.645)	0.428(0.400-0.460)**
	d18:1/C22:0	1.442(1.294-1.596)	1.012(0.930-1.024)**
	d18:1/C24:0	2.574(2.377-2.648)	2.076(1.983-2.123)**
	d18:1/C24:1	5.063(4.532-5.385)	2.921(2.473-3.127)**

Values are median (pmol/mg of tissue) and interquartile range; n=8 per group; Wilcoxon signed rank test for paired samples

^{ns} -p > 0.05; * -p ≤ 0.05; **- p ≤ 0.01 vs $HFD_{(+Acsl1)}$ gastrocnemius.
