# Supplementary material for: shRNA-mediated down-regulation of Acsl1 reverses skeletal muscle insulin resistance in obese C57BL6/J mice
Source: PLoS One. 2024 Aug 23;19(8):e0307802. doi: 10.1371/journal.pone.0307802 (PMC11343424; doi:10.1371/journal.pone.0307802)
Supplement: Attachment — Submitted filename: Response to Reviewers.docx [file pone.0307802.s012.docx]

**Reviewer #1:** Roszczyc-Owsiejczuk et al., investigated the role of ACSL1 in skeletal muscle in regulating insulin resistance under high fat diet using mouse model. It is an interesting topic. However, I have some concerns about the experiment design. My comments are listed below:

**Reply:** We thank the Reviewer for his effort to improve our manuscript. We hope, that updated manuscript will gain the Reviewer recommendation for publication.

1. The introduction is too long. Please reorganize this part and make it more concise.

**Reply:** To improve its readability, we shortened and re-organized the introduction as requested.

2. What is the age and sex of the mice used in this experiment, please clarify. Did you check the food intake of the mice? Did you check muscle mass and muscle histology for all the groups? If lipid metabolism has been affected, it is possible to see the difference of lipid droplet staining in skeletal muscle between -shRNA and +shRNA ACSL1 group under the HFD condition.

**Reply:** The study was performed on male C57BL/6J mice of 6 weeks of age at the beginning of the experiment. Mice were fed for 8 weeks with HFD to induce whole-body insulin resistance. Animals were euthanized at the age of 14-15 weeks. Above information was previously included in “Materials and Methods” section. We introduced “Animals” paragraph and put this information at the beginning of the “Methods” secretion, as previously it was somehow hidden in the experiment design details.

Mice were housed at 4 individuals per cage, according to IACUC-accepted procedure. Unfortunately, individual food consumption monitoring was not possible.

The weight of non-silenced muscle and ACSL1-silenced muscle in HFD-fed animals was indifferent between both legs and presented physiological morphology (Supplement Figure S2). During our initial small-scale experiment to refine electroporation technique, the muscle displayed normal histology under HE (hematoxylin/eosin) stain, except small necrotic area directly around needle injection. This was observed similarly in both non-silenced and Acsl1-silenced muscle.

Regarding lipid droplet stain, unfortunately we did not perform appropriate preparations, due to the fact, that all the gastrocnemius muscle tissues from experimental animals were pulverized in LN_2_ prior to all the subsequent measurements. Nevertheless, the drop in muscle TAG accumulation (now shown in Figure 3A2) in HFD_(-ACSL1)_ gastrocnemius as compared to HFD_(+Acsl1)_ gastrocnemius can be a good indicator of possible decrease in lipid droplet content in Acsl1 down-regulated muscle.

3. The most confusing part is the experiment design. Why did not have LFD -shRNA ACSL1 group? It is not clear how many groups you used in the current study based on your description in materials and methods. I believe you have 4 groups LFD, HFD and HFD-shRNA ACSL1, HFD+shRNA ACSL1 (Although the last two groups were actually left and right leg of the same mouse) based on your Figure S1. However, you did not mention HFD group in your materials and methods. At least to me, it is not reasonable to compare LFD with both legs treated with scrambled shRNA with only one leg treated with scrambled shRNA and the other leg treated with shRNA ACSL1. If in the LFD condition you also perform the same treatment like the HFD it is acceptable. Then it could be a 2x2 factorial experiment design.

**Reply:** Thank you for this valuable comment. We agree that performing statistical analysis between those 3 tissues (LFD_(+Acsl1)_, HFD_(+Acsl1)_ and HFD_(-Acsl1)_ gastrocnemius) using ANOVA statistics was not optimal. We changed both the data presentation and statistical analysis. In our study, non-silenced gastrocnemius from LFD-fed mice (LFD_(+Acsl1)_ muscle) served as a baseline, insulin-sensitive tissue for non-silenced insulin-resistant gastrocnemius form HFD-fed mice (HFD_(+Acls1)_ muscle). In the revised manuscript we present this comparison on separate figures and use non-parametric Wilcoxon rank sum test for unpaired samples to establish significance. For within-animal comparison between non-silenced HFD_(+Acls1)_ muscle and contralateral silenced HFD_(-Acsl1)_ muscle, we employed non-parametric Wilcoxon signed rank test for paired samples. We also present the results of this comparison on separate figures. In both unpaired- and paired comparisons we opted for non-parametric Wilcoxon test over respective t-test due to small sample size. Although comparisons using Wilcoxon statistics gave higher p-values than t-test, significance was retained. We also modified the results section to clarify the confusion regarding the experimental groups and their comparisons.

Our rationale to obtain both non-silenced and silenced gastrocnemius from the same animal with HFD-induced insulin resistance was to exclude the influence of other factors, which could affect skeletal muscle insulin resistance. Thus the differences in gastrocnemius insulin action and bioactive lipid content between ACSL1 silenced (HFD(-Acsl1) and non-silenced (HFD(+Acsl1) muscle can be only attributed to ACSL1 modulation. The non-silenced LFD(+Acsl1) muscle was introduced as a baseline, insulin sensitive tissue, to observe the effects of HFD feeding on muscle lipid metabolism and insulin sensitivity in silenced HFD(+Acsl1) muscle.

Moreover, we updated Figure S2 to better present our experimental design.

We hope that updated manuscript fully addresses those important issues.

4. Based on your results, high fat diet could increase the protein expression of ACSL1. Even though you knockdown ACSL1 but the protein level is still higher than LFD condition which suggests that diet may have a stronger effect than ACSL1 itself. That is also why it is necessary to knockdown ACSL1 in the LFD condition to see the efficiency of its knockdown. Besides, you noticed the insulin signaling pathway has been altered in ACSL1 knockdown condition, but overall systemic metabolism did not change based on your glucose tolerance test and insulin tolerance test. This also suggest that other organs such as liver or adipose tissue might be affected in the HFD condition.

Reply: We are grateful for this important remark. In our experiment we achieved modest down-regulation of Acsl1 at protein level. Much stronger effect was achieved at mRNA expression level. We hypothesize, that due to very slow turnover rate of muscle proteins, the silenced gastrocnemius could still retain Acsl1 protein from pre-electroporation stage, yet with decreased biological activity. This is evidenced by significant drop in LCACoA content, most visible for 16- and 18-carbon CoAs (preferred Acsl1 substrate/product fatty acids chain length).

Regarding the systemic effects, we chosen local single gastrocnemius Acsl1 silencing in HFD-fed insulin resistant mice especially not to affect whole-body insulin resistance. The affected muscle volume was too small to modify systemic FA metabolism. Thus all the beneficial effects on muscle insulin sensitivity could be attributed solely to the local Acsl1 silencing, not to the modification of other factors. We ruled-out Acsl1 knock-out approach, as it modifies whole-body FA metabolism blurring the muscle-specific mechanisms of insulin resistance. In the Materials for Reviewers, we present Figure S1 and Table S1, which compares anthropometric values of HFD animals with no silencing (from our initial experiments, HFD1 animals) with values of HFD animals with single gastrocnemius silencing (HFD_(-Acsl1)_). Data indicates, that local Acsl1 silencing had no observable effects at systemic level.

We agree, that other organs e.g. liver and adipose tissue are crucial in the multifaceted mechanisms of systemic insulin resistance. Our experiment aimed at elucidation of muscle-specific mechanisms of diet induced IR. We also performed a follow-up project, which employed liver-specific shRNA-mediated silencing of lipid-metabolism enzymes via hydrodynamic gene delivery technique, which results are currently being prepared for publication.

5. It will be nice to do lipidomics using skeletal muscle tissue and plasma which can help understand the role of ACSL1 in skeletal muscle.

**Reply:** The non-targeted lipidomic approach combined with stable isotope labeling and LC/MS/MS is a powerful tool which could shed the light on the fate of fatty acids on Acsl1-silenced tissue. Regarding the current study, we had no access to this methodology. Nevertheless, our targeted LC/MS/MS-based technique cover significant portion of bioactive lipidome, especially molecular species of DAG and Cer with proven biological activity.

**Reviewer #2: Summary**
This paper demonstrates that ablation of the lipid-activating enzyme Acsl1 in skeletal muscle is beneficial and ameliorates insulin resistance. The authors utilize single leg electroporation to deliver plasmids against Acsl1 to one leg and show that, in a high fat-diet context, inhibition of the fatty acid conversion to the acyl CoA-derivatives is able to rescue the insulin resistant phenotype observed in the contralateral leg muscle. This is relevant, given that the uptake of free fatty acids is not reduced, but without activation into acyl-CoA, lipid synthesis byproducts such as DAG and Sphingolipids are not produced. This is confirmed by a reversal of the insulin resistant phenotype. Altogether, Acsl1 ablation is sufficient to rescue the insulin resistant muscle.

**Reply:** We are grateful for the valuable comments by Reviewer, which allowed us to improve our manuscript. We hope, that employed corrections will answer all the important questions raised by the Reviewer and fulfill the standards for the recommendation for publication.

**Major comments**
The paper is clear and the phenotype reported is relevant to understand how free fatty acid activation by Acsl1 is at the crossroads of fatty acid oxidation and lipid synthesis in muscle. My major concern with the current paper is the statistical analysis. As described in the methods section (Page 6, line 128; Supplement Figure S2), the transfection of the Acsl1 snRNA was done in the “Left gastrocnemius muscle of high-fat diet mice was electroporated with active shRNA plasmid… yielding silenced HFD(-Acsl1) gastrocnemius, while the contralateral, right hindlimb gastrocnemius within the same animal was transfected with scrambled shRNA, yielding HFD(+Acsl1) gastrocnemius.” Additionally, the authors provide a schematic to describe the experimental design: 16 mice were used for the transfection studies, where only one group (n=8) of HFD-fed mice were transfected on the each leg with different shRNAs. Thus, this presents difficulties when performing statistical tests. Given that only HFD mice, and not LFD, have a left (-Acsl1- gasctrocnemius, comparing to LFD is not a valid or direct control. This is because the LFD group received scrambled shRNA on both legs, and no Acsl1- shRNA. Thus, the group comparison is not possible. Additionally, the authors treat the HFD-fed legs as different and independent groups (showing 3 groups total). It is misleading to display data in this way. Investigators should be discouraged from visualizing within animal and between animal comparisons on the same figure. These data could be compared such as with paired statistics or two separate unpaired tests, all with correction for Family wise error. Additionally, tests should be corrected for multiplicity using Bonferroni or similar correction factor. This is important, given that previous papers that are cited for single leg transfections make use of such statistics. This treatment has systemic effects, and thus the data from left leg and right leg are dependent upon each other, and should be a paired. Investigators should re-run statistics and adjust all figures and tables to avoid comparison between each leg of the same mouse and across groups within the same graph.

**Reply:** We are grateful for pointing us towards this issue. We agree that performing statistical analysis between 3 tissues (LFD+, HFD+ and HFD- gastrocnemius) using ANOVA statistics was not optimal. According to the Reviewer comments, we changed both the data presentation and statistical analysis. In the updated manuscript, significance between gastrocnemius from LFD-fed animals (sham-electroporated LFD(+Acsl1) muscle) and matching gastrocnemius from HFD-fed animals (sham-electroporated HFD(+Acsl1) muscle) was tested with non-parametric Wilcoxon rank sum test for unpaired samples and the data is presented on separate figure panels. For within-animal comparison between sham-electroporated HFD(+Acsl1) muscle and silenced HFD(-Acsl1) muscle, we employed non-parametric Wilcoxon signed rank test for paired samples and similarly, we present the results on separate figures. In both unpaired- and paired comparisons we opted for non-parametric Wilcoxon test over respective t-test due to our small sample size and higher power of the Wilcoxon test. Although comparisons using Wilcoxon statistics gave higher p-values than that of t-test (or our original ANOVA), significance was retained. We also modified the results section and updated Figure S2 to clarify the confusion regarding the experimental groups.

We hope that updated manuscript fully addresses those important issues.

Given only two groups are examined in the current paper, how were the metabolic tests performed to show 3 groups?

Reply: We understand that the Reviewer is pointing us towards HFD_1_ animals from original Supplement file S1 (anthropometric and metabolic tests). This particular group of HFD-fed animals had gastrocnemius in both legs sham-electroporated with non-coding plasmid. We measured only anthropometric/metabolic parameters in those animals. Anthropometric values from this group show, that there is no systemic alternations between those animals and the animals with Acsl1 down regulation in single gastrocnemius (HFD group which yields both +Acsl1 and -Acsl1 tissue). This is expected, as down-regulation of Acsl1 in single gastrocnemius is unlikely to exert noticeable effects at systemic level (e.g plasma fatty acids). Contrary to pharmaceutical intervention, whole-body knock out or muscle-specific knock-out, local silencing has no systemic effects. Thus the improvement in muscle-level insulin action in Acsl1-down regulated muscle can be attributed only to the modulation of Acsl1 and its LCACoA product but no other systemic factors.

We are sorry, that introduction of this particular set of animals led to misunderstanding. As introduction of those animals led to confusion, we removed the data from HFD_1_ animals from the revised manuscript.

The authors demonstrate that acyl-CoAs, diacylglycerides, and sphingolipids are reduced in the Acsl1- muscle, and further show that this is followed by a paradoxical increase in CPT1B, but a reduction in β-oxidation proteins (HADHA, ACADVL, ACADL) and acyl-carnitines. However, no changes in FFA are observed between the HFD left Acsl1- and right Acsl1+ gastrocnemius muscles or fatty acid transporters. What is the fate of these FFA? What is the interpretation for such results? This should be added to the discussion.

**Reply:** We did not analyze intracellular free fatty acids in the muscle tissue. The FFA data from previous version of Supplement Table S1 (now in Materials for Reviewers) refers to the systemic parameters, e.g plasma free fatty acids. Regarding the fate of intramuscular fatty acids in Acsl1-silenced tissue, we hypothesize, that since FFA activation to Acyl-CoA is decreased, excess of plasma FFA do not enter metabolic pathways which lead to lipid synthesis or β-oxidation. Decrease in muscle mitochondrial β-oxidation was observed in the muscle of ACSL1 knock out mice fed low-fat diet. As both Acsl1 and fatty acids transporter FATP1 co-localize and create complex in plasma membrane and mitochondrial outer membrane, mitochondrial Acyl-CoA uptake could be significantly decreased, as evidenced by significant decrease in acyl-carnitines in Acsl1-silenced muscle. The decrease in the Acyl-CoA flow through metabolic pathways could be the reason for paradoxical up-regulation of both the FAPBpm membrane fatty acids transporter and CPT1 protein. Yet with decreased metabolic capture of FFA into LCACoA, free fatty acids could diffuse out of the cell by flip-flop mechanism. We discussed possible fate of FA in the revised discussion.

Could the increase in the right Acsl1+ gastrocnemius be a compensation for reduced fatty acid activation by the left Acsl1- muscle? Do other muscles experience this? Are the changes observed in the Acsl1+ gastrocnemius similar to a HFD with both Acsl1+ legs?

**Reply:** Compensatory up-regulation of Acsl1 in non-silenced muscle would require existence of feedback mechanism. Changes in plasma fatty acids content and composition could act as relay signal which in turn could affect Acsl1 expression in other tissues. Nevertheless we did not observe any significant changes in anthropometric parameters and plasma FFA between HFD-fed mice without gastrocnemius Acsl1 silencing and those with local silencing of Acsl1.

The beneficial effects of Acsl- ablation are important and increase our understanding of how fatty acid activation can be utilized to restore insulin sensitivity to the muscle. What is the effect on energy metabolism? Other cited papers show that these muscles increase the use of glucose and amino acids. This should be expanded with the GLUT4 and glucose uptake findings.

Reply: Thank you for this valuable suggestion. We included in the discussion paragraph regarding the energy sources of Acsl1-silenced muscle.
