# Supplementary material for: shRNA-mediated down-regulation of Acsl1 reverses skeletal muscle insulin resistance in obese C57BL6/J mice
Source: PLoS One. 2024 Aug 23;19(8):e0307802. doi: 10.1371/journal.pone.0307802 (PMC11343424; doi:10.1371/journal.pone.0307802)
Supplement: Attachment — Submitted filename: Response to Reviewers 10_06_2024.docx [file pone.0307802.s013.docx]

Manuscript: PONE-D-23-38615R1

shRNA-mediated down-regulation of Acsl1 reverses skeletal muscle insulin resistance in obese C57BL6/J mice

**Response to Reviewers**

Dear Herve Le Stunff

Thank You for giving us the opportunity to submit a revised version of the manuscript for possible publication in the PLOS ONE. We appreciate the time and effort that You and the Reviewers have dedicated to providing the feedback on our manuscript. We are grateful for the insightful comments and valuable improvements to our paper. We have incorporated both the Editor, Editorial Office and the Reviewers' suggestions. Please find below our point-by-point response to the Reviewers’ comments and concerns.

**Response to Reviewer 1:**

**Comment from Reviewer 1:**The aim of this study was to determine whether long-chain acyl-CoA (LCACoA) and the enzyme that synthesises them, long-chain acyl-CoA synthetase (Acsl1), can modulate muscle insulin sensitivity in mice. The authors KO'd Acsl1 by electroporating a shRNA directed against Acsl1 into the gastrocnemius muscle of mice, while the muscle of the other leg was electroporated with a control shRNA. The animals were then fed a fatty diet for 8 weeks. The authors show that reduced expression of ACSL1 induces a reduction in LCACoA, ceramides and diacylglycerols, and improves insulin sensitivity compared with control muscle. They also observed a reduction in mitochondrial fatty acid metabolism. The results are interesting and show the involvement of long-chain fatty acids in the development of muscle insulin resistance in mice. However, a number of important details raise questions and need to be clarified for the data to be unequivocal.

**Author’s response*:*** We would like to thank the Reviewer for providing valuable comments that have allowed us to improve our manuscript. Please find below our detailed response.

**Comment from Reviewer 1:**An important detail in the introduction to the article: the authors state that ceramides inhibit the muscular insulin response by activating PP2A phosphatase, which acts negatively on Akt (line 62-63). However, it has been clearly shown previously that in muscle cells ceramides act via activation of PKCz (Powell et al, 2003, Fox et al, 2007, Hajduch et al, 2008, Mahfouz et al, 2014). The authors cannot ignore this fact.

**Author’s response:** Thank You for bringing this to our attention. We have carefully revisited the Introduction section of the manuscript, and made the necessary revisions to address Reviewers concern regarding ceramide-mediated PKCζ activation.

**Comment from Reviewer 1:**The major problem with the article is in Figure 1, where the authors show that electroporation worked and that shRNA decreased Acsl1 expression in the gastrocnemius of mice. However, not everything seems very clear. In Figure 1B2, the authors show a decrease in Acsl1 expression in the muscle in response to shRNA. This decrease appears to be statistically real. They also state that they did not observe any compensation with the other Acsl isoforms (Supplementary Figure 5). However, when we look at this last figure, the results do not seem so clear-cut. Indeed, the error bars are often larger than the expression levels of the isoforms. As a result, of course, there is no statistical difference between electroporated and non-electroporated. I don't understand how there can be so much variation between mice (8 mice) concerning the expression of these Acsl isoforms and, on the contrary, so little concerning Acsl1 (figure 1B2). I think it's necessary for the authors to redo their PCRs because it's obvious that something isn't right.

**Author’s response:** We agree that variability in mRNA expression analysis of other Acsl isoforms was indeed excessive. As recommended, we re-analyzed all Acsl isoforms gene expression (from Acsl1 to Acsl6) and the data is presented in updated Figure 1 and Supplement 1 Figure S5. The new PCR measurements were performed in a single batch by the same technician.

**Comment from Reviewer 1:**Another major problem concerns the blots shown in all the figures. The authors have cut the bands they were interested in and put them side by side in the figures because the original blots contain many unused bands. This doesn't seem right to me. First of all, I'd like to know what all these unused strips in all the blots correspond to. What's more, for each point, there are 4 strips in the uncut blots. However, the authors only show 2 in the final figures. How did they choose the final bands? For the quantifications, did they quantify the 4 bands or the two bands? This is a real problem for me. Especially concerning the Acsl1 bands. It seems to me that the contrast has been changed between the two bands corresponding to HFD(+Acsl1) presented in figure 1 and the same bands in the original blot. For me, the authors should migrate again their samples side by side so that they can present them properly. I must admit that as it stands, there may remain some doubt as to the value of the data.

**Author’s response:** Thank You for pointing us towards this inconsistency. In response to Your concerns and to ensure the accuracy of our data presentation, we conducted additional WB analysis for the Acsl1 protein and have included the results in Figure 1 B1-B2. Regarding additional lanes in the original blots, those belong to other experimental groups from the larger study. Those included muscle samples with silencing of other lipid metabolism-related genes, such as CerS or GPAT (data already published). Our desire was to perform the exemplary WBs for all the experimental groups in the single, large, 26-lane blot, thus reducing blot-to-blot variability. The updated Supplement 2 shows which samples were taken for Acsl1-related study.

**Comment from Reviewer 1:**Another point to raise, are you sure the data is statistically different in Figure 1B1? Looking at the error bars, I have my doubts. Is Acsl1 expression indeed increased in muscle in response to HFD? The authors should show the dispersion of points in all their results so that variations between experimental conditions can be seen. I find the data in the paper interesting but their importance depends on the results of Figure 1 which poses a problem for me.

**Author’s response:** Thank You for Your feedback regarding the presentation of our data. Regarding the Acsl1 expression at both the protein and mRNA levels, the Figure 1 now contains the data from re-analyzed samples (as mentioned earlier). Updated figures include both the medians (as bars), interquartile ranges (as whiskers) and individual data points (as appropriate markers). Additionally, we re-analyzed all the data using Student’s t-statistics in both the unpaired (for LFD_(+Acsl1)_ vs HFD_(+Acsl1)_ comparison) and paired (for HFD_(+Acsl1)_ vs HFD_(-Acsl1)_ comparison) versions (not shown in the manuscript) to confirm the findings. We noted similar, or - in most cases - higher significance results (smaller p-values) in the case of Student’s t-test than those observed in the case of Wilcoxon non-parametric test. Only in the case of SCA-Car values from LFD_(+Acsl1)_ vs HFD_(+Acsl1)_ comparison, the parametric t-test gave non-significant results as compared to Wilcoxon test. Although individual data-point distribution indeed suggests lack of significance in the abovementioned comparison, we believe that the use of non-parametric statistics is more applicable for our study due to small n-numbers which do not allow for proper testing of data normality, and thus exclude employment of parametric tests such as paired or un-paired Students t-test. We are hesitant to selectively present t-test significance results for SCA-Car data with other measurements tested by the Wilcoxon method, unless the Reviewer will insist otherwise. We hope that the updated data presentation will sufficiently address the Reviewer questions.

**Reviewer #4:** In this article, the authors explore the impact of ACSL1 on insulin resistance in skeletal muscle within the context of diet-induced obesity (DIO) in C57Bl6/J mice. Despite the unclear connection between ACSL1, fatty acid beta oxydation, and lipid metabolism concerning ectopic lipid accumulation, the authors employed electroporation to deliver a shRNA plasmid targeting ACSL1 to one gastrocnemius muscle while the other gastrocnemius received a sham shRNA plasmid.
The authors highlight modifications in ACSL1 expression, fatty acid beta-oxydation proteins, bioactive lipids contents, and insulin signaling during DIO. They demonstrate that inhibiting the conversion of fatty acids into SCA-coa and LCA-coa using an shRNA plasmid targeting ACSL1 can mitigate the harmful effects of DIO. Specifically, they report normalization of ACSL1 expression, restoration of SCA-coa and LCA-coa, DAG, and cermides levels, as well as the recovery of beta-oxidation protein expression in their experimental conditions.

Major comments:
1. The authors chose to carry out their study using the C57Bl6/J mouse strain instead of C57Bl6/N. It is essential to justify this decision considering the references below:
o Kaku K, Fiedorek FT Jr, Province M, Permutt MA: Genetic analysis of glucose tolerance in inbred mouse strains: evidence for polygenic control. Diabetes 37:707–713, 1988.^1^
o Kooptiwut S, Zraika S, Thorburn AW, Dunlop ME, Darwiche R, Kay TW, Proietto J, Andrikopoulos S: Comparison of insulin secretory function in two mouse models with different susceptibility to beta-cell failure. Endocrinology 143:2085–2092, 2002.
o Freeman HC, Hugill A, Dear NT, Ashcroft FM, Cox RD: Deletion of nicotinamide nucleotide transhydrogenase: a new quantitative trait locus accounting for glucose intolerance in C57BL/6J mice. Diabetes. 2006 Jul;55(7):2153-6.

The authors’ DIO effectively induced glucose intolerance and insulin resistance, as evidenced by comparisons with the LFD group, and their selection of C57Bl6/J strain does not undermine the study’s validity.

**Author’s response:**

We acknowledge the significance of the provided references, which document differences in both the genotype and phenotype between different mice strains. We agree that C57BL/6J-specific mutation in Nnt gene leading to the expression of non-functional mitochondrial nicotinamide nucleotide transhydrogenase, may affect mitochondria-specific features of cellular metabolism, especially NADPH biosynthesis, glutathione reduction and free radical detoxification (1,2) Although the most significant phenotypic variation between C57BL6/J and other BL6 strains is β-cell energy metabolism and insulin release, the effect of Nnt deletion seem to be moderate in nature (3,4). Control C57BL6/J mice lacking functional Nnt gene housed on standard, low-fat diet are still significantly more glucose-tolerant and insulin sensitive from their high-fat diet-fed counterparts (5), which validates the use of C57BL/6J strain in the studies on diet-induced insulin resistance.

The major factor influencing our choice of C57BL/6J mouse strain over other well established BL6 and DBA/2 strains was both the documented susceptibility to metabolic disorders under HFD feeding and its widely recognized status in insulin resistance and T2D research (6,7). C57BL6/J strain's predisposition to develop insulin resistance makes it a preferred model for studying metabolic disorders induced by high-fat diets (8). Currently C57BL/6J is the most popular BL6 strain used in diabetes-related studies, according to PubMed. Moreover, the significant body of research, which employed modern high-throughput techniques such as genomics, transcriptomics and proteomics was collected using this particular strain, as it was originally used as a reference organism in Mouse Genome Project initiative (9,10). Consequently, the utilization of the C57BL/6J mice, with its established predisposition to glucose intolerance and reduced insulin secretion under high-caloric intake, offers a robust model for investigating metabolic disorders. To address the selection of C57BL6J mice in our study we updated the Introduction section of the manuscript.

1. Moon D. O. (2023). NADPH Dynamics: Linking Insulin Resistance and β-Cells Ferroptosis in Diabetes Mellitus. International journal of molecular sciences, 25(1), 342. doi.org/10.3390/ijms25010342.
2. Ronchi JA, Figueira TR, Ravagnani FG, Oliveira HC, Vercesi AE, Castilho RF. A spontaneous mutation in the nicotinamide nucleotide transhydrogenase gene of C57BL/6J mice results in mitochondrial redox abnormalities. Free Radic Biol Med. 2013;63:446-456. doi:10.1016/j.freeradbiomed.2013.05.049.
3. Close AF, Chae H, Jonas JC. The lack of functional nicotinamide nucleotide transhydrogenase only moderately contributes to the impairment of glucose tolerance and glucose-stimulated insulin secretion in C57BL/6J vs C57BL/6N mice. Diabetologia. 2021;64(11):2550-2561. doi:10.1007/s00125-021-05548-7.
4. Attané C, Peyot ML, Lussier R, et al. Differential Insulin Secretion of High-Fat Diet-Fed C57BL/6NN and C57BL/6NJ Mice: Implications of Mixed Genetic Background in Metabolic Studies. PLoS One. 2016;11(7):e0159165. Published 2016 Jul 12. doi:10.1371/journal.pone.0159165.
5. Fisher-Wellman KH, Ryan TE, Smith CD, et al. A Direct Comparison of Metabolic Responses to High-Fat Diet in C57BL/6J and C57BL/6NJ Mice. Diabetes. 2016;65(11):3249-3261
6. Montgomery MK, Hallahan NL, Brown SH, et al. Mouse strain-dependent variation in obesity and glucose homeostasis in response to high-fat feeding. Diabetologia. 2013;56(5):1129-1139;
7. Nguyen-Phuong T, Seo S, Cho BK, Lee JH, Jang J, Park CG. Determination of progressive stages of type 2 diabetes in a 45% high-fat diet-fed C57BL/6J mouse model is achieved by utilizing both fasting blood glucose levels and a 2-hour oral glucose tolerance test. PLoS One. 2023;18(11):e0293888).
8. Collins S, Martin TL, Surwit RS, Robidoux J. Genetic vulnerability to diet-induced obesity in the C57BL/6J mouse: physiological and molecular characteristics. Physiol Behav. 2004;81(2):243-248).
9. Mouse Genome Sequencing Consortium, Waterston RH, Lindblad-Toh K, et al. Initial sequencing and comparative analysis of the mouse genome. Nature. 2002;420(6915):520-562. doi:10.1038/nature01262.
10. Sarsani VK, Raghupathy N, Fiddes IT, et al. The Genome of C57BL/6J "Eve", the Mother of the Laboratory Mouse Genome Reference Strain. G3 (Bethesda). 2019;9(6):1795-1805. Published 2019 Jun 5. doi:10.1534/g3.119.400071.

2. The authors do not specify whether CPT2 is altered under their experimental conditions or if a compensatory mechanism occurs in HFD_(+Acsl1)_ and HFD_(-Acsl1)_.

**Author’s response:** Thank You, Reviewer, for Your insightful comment. Based on the literature, we selected CPT1b (membrane carnitine palmitoyltransferase 1, muscular isoform) as a marker enzyme for mitochondrial fatty acids import. This isoform is widely recognized as a crucial rate-limiting enzyme that regulates the rate of long-chain fatty acid entry into the mitochondrial β-oxidation (1). Therefore, we opted to focus on presenting CPT1b protein expression in our analysis, considering its significance to our research objectives. Consequently, we determined that performing additional CPT2 protein analysis would be excessive, due to the limited amount of remaining material available for experimentation.

1. Bruce CR, Hoy AJ, Turner N, et al. Overexpression of carnitine palmitoyltransferase-1 in skeletal muscle is sufficient to enhance fatty acid oxidation and improve high-fat diet-induced insulin resistance. Diabetes. 2009;58(3):550-558).

3. In Figure 4, the authors measure the phosphorylation of Akt/PKB by assessing the phosphorylation of Akt Ser473 using western blotting. It would be valuable to know if they also conducted western blot analysis with an antibody targeting Thr308-Akt since pAKT/PKB requires activation on both sites (Thr308 and Ser473) for full signaling pathway activity.

**Author’s response:** We appreciate Your valuable comment. Following the Reviewer's suggestion, we analyzed Akt Thr308 phosphorylation. The results presented in Figure 4 show, that Acsl1 partial ablation is connected with AKT phosphorylation at both the Thr308 and Ser473 sites. This finding further confirms the impact of muscle Acls1 downregulation on the activity of insulin signaling pathway.

Minor comments:
1. The figures in the revised submission appear to have formatting issues, making them difficult to read, particularly Figures 2 and 4. It is crucial to upload them in higher resolution.

**Author’s response:** In the revised submission we have addressed the figure formatting issues by changing their appearance, increasing the size of exemplary WBs and uploading figures in higher resolution. We hope this will ensure improved readability of our data. Thank You for bringing this to our attention.

2. The references’ section (line 422) appears to use two different formats for presenting references. Kindly standardize the format.

**Author’s response:** We have taken steps to standardize the format, ensuring uniformity throughout. Thank You for pointing us towards this inconsistency.

3. In the introduction and discussion, the authors inconsistently describe lipid species using either the “C16:0” notation or “18-carbon Cer.” A more consistent presentation would improve readability.

**Author’s response:** By 18-carbon length Cer we meant all the Cer species acylated with 18-carbon length fatty acids, both saturated (C18:0-stearic) and unsaturated (C18:1-oleic, C18:2-linoleic, C18:3-linolenic). We acknowledge, that our description could be misleading and we will ensure to name the molecular species of lipids directly (e.g C18:0-Cer, C18:1-Cer, C18:0-CoA etc.) throughout the introduction and discussion sections. Thank You for pointing us toward this inconsistency.
